# Neurological morphofunctional differentiation induced by REAC technology in PC12. A neuro protective model for Parkinson’s disease

**DOI:** 10.1038/srep10439

**Published:** 2015-05-15

**Authors:** Margherita Maioli, Salvatore Rinaldi, Rossana Migheli, Gianfranco Pigliaru, Gaia Rocchitta, Sara Santaniello, Valentina Basoli, Alessandro Castagna, Vania Fontani, Carlo Ventura, Pier Andrea Serra

**Affiliations:** 1 Department of Biomedical Sciences, University of Sassari, 07100, Sassari, Italy; 2 Laboratory of Molecular Biology and Stem Cell Engineering - National Institute of Biostructures and Biosystems, 40121, Bologna, Italy; 3 Department of Regenerative Medicine, Rinaldi Fontani Institute, 50144, Florence, Italy; 4 Research Department, Rinaldi Fontani Foundation - NPO, 50144, Florence, Italy; 5 Stem Wave Institute for Tissue Healing (SWITH), Gruppo Villa Maria and Ettore Sansavini Health Science Foundation NPO, 48022, Lugo, Italy; 6 Department of Clinical and Experimental Medicine, University of Sassari, 07100, Sassari, Italy

## Abstract

Research for the use of physical means, in order to induce cell differentiation for new therapeutic strategies, is one of the most interesting challenges in the field of regenerative medicine, and then in the treatment of neurodegenerative diseases, Parkinson’s disease (PD) included. The aim of this work is to verify the effect of the radio electric asymmetric conveyer (REAC) technology on the PC12 rat adrenal pheochromocytoma cell line, as they display metabolic features of PD. PC12 cells were cultured with a REAC regenerative tissue optimization treatment (TO-RGN) for a period ranging between 24 and 192 hours. Gene expression analysis of specific neurogenic genes, as neurogenin-1, beta3-tubulin and Nerve growth factor, together with the immunostaining analysis of the specific neuronal protein beta3-tubulin and tyrosine hydroxylase, shows that the number of cells committed toward the neurogenic phenotype was significantly higher in REAC treated cultures, as compared to control untreated cells. Moreover, MTT and Trypan blue proliferation assays highlighted that cell proliferation was significantly reduced in REAC TO-RGN treated cells. These results open new perspectives in neurodegenerative diseases treatment, particularly in PD. Further studies will be needed to better address the therapeutic potential of the REAC technology.

Previous studies, conducted using the radio electric asymmetric conveyor (REAC) technology, have shown that this technology is able to induce neurogenic cell differentiation both in cultures of murine embryonic cells^1^ and in differentiated human cells, such as fibroblasts^2^ and adipocytes^3^. In addition, the REAC technology has been shown to effectively counteract cell aging^4^,^5^,^6^, a process often related to neurodegenerative diseases as Parkinson’s disease (PD). To better dissect and understand the potential of the REAC treatments in PD, we chose a PC 12 cellular model. This model was widely used to study neuron functions and to understand the physiology of central dopamine (DA) neurons. Therefore, we think this study can provide useful information and pave the way to future possible application of REAC technology in the treatment of PD.

## Results

### REAC TO-RGN exposure primes cell commitment toward a neurogenic phenotype

**Figure 1:**
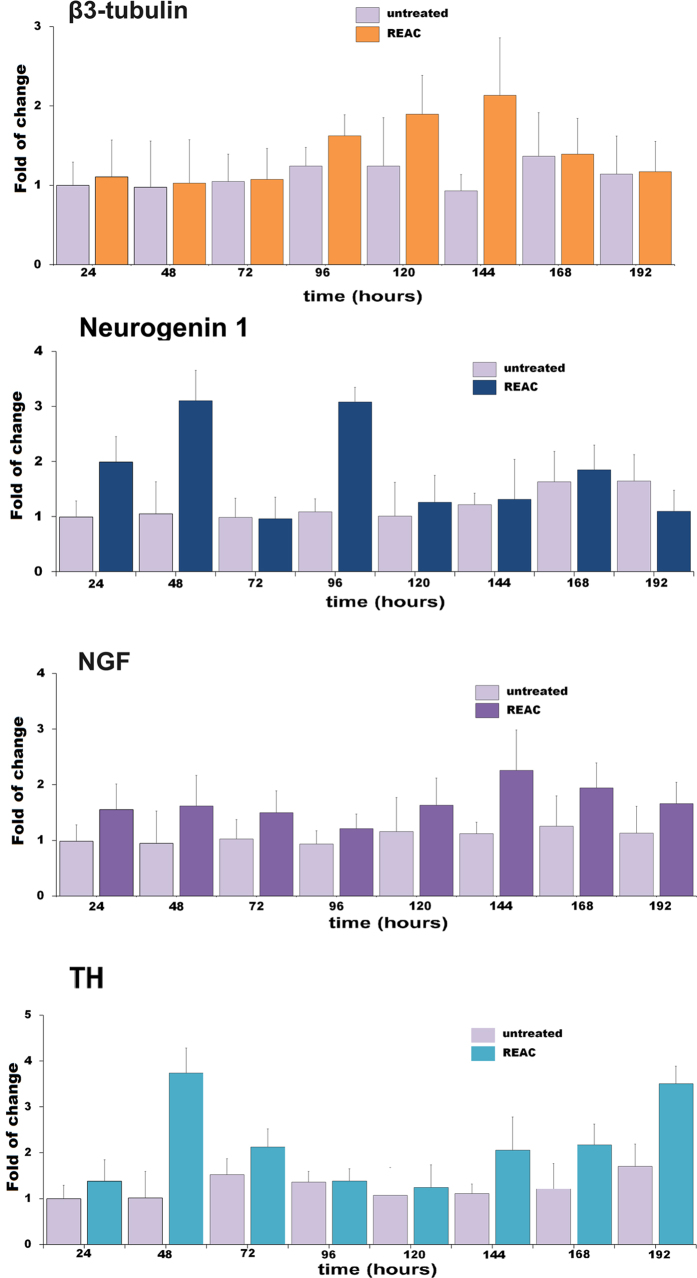
Effect of REAC TO-RGD treatment on the expression of neuritogenesis regulating genes in PC12 cells. Cells were exposed from 1 (24 h) to 8 days (192 h) in the absence or presence (darker bars) of REAC TO-RGN. The amounts of β3 tubulin, neurogenin-1 NGF and tyrosine hydroxylase (TH) mRNA from REAC TO-RGN treated or untreated cells were normalized to GAPDH, and the mRNA expression of REAC TO-RGN treated cells was plotted at each time point as fold of change relative to the expression in PC12 untreated cells cultured for 24 hours after plating (named ND) defined as 1 (mean ± S.E.; n = 6). All the REAC TO-RGN treated cells at each time point were significantly different from each control untreated cells (mean ± S.E.; n = 6; P < 0.05).

**Figure 2:**
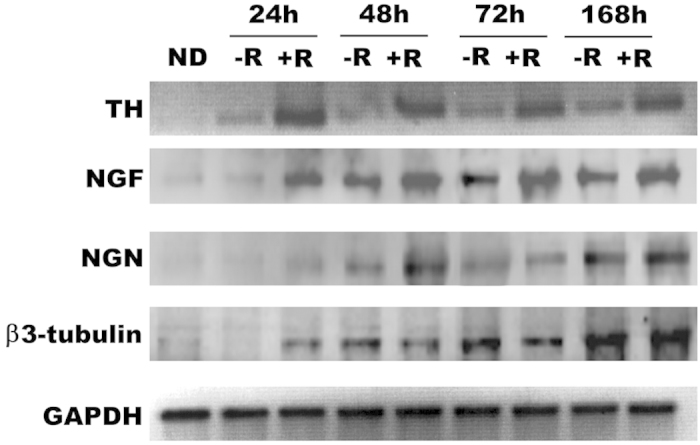
Effect of REAC TO-RGD treatment on the expression of neuritogenesis associated proteins in PC12 cells. Total lysates were isolated from PC12 cells, exposed for 24, 48, or 72 hours and for 7 days in the absence (−R) or presence of REAC (+R). Samples were analyzed by Western blot, using antisera against tyrosine hydroxylase (TH), NGF, neurogenin 1 (NGN), ß-3 tubulin and GAPDH. The sizes of the bands were determined using prestained marker proteins. The data presented are representative of five separate experiments. Two different membranes were incubated with antirabbit (NGF ,GAPDH) or antimouse (β Tubulin isotype III) or antigoat (Neurogenin).

Figure 1 shows the expression of the neurogenic phenotype associated genes β3-tubulin and neurogenin-1, and nerve growth factor (NGF) in PC12 cells exposed to REAC TO-RGN for 24 h (1 day) to 192 h (8 days); β3-tubulin and neurogenin-1 were expressed both in control and in REAC TO-RGN treated cells. But after 96 hours REAC TO-RGN treated cells exhibited significantly higher levels of β3-tubulin, as compared to control cells; on the other hand neurogenin-1 mRNA levels were significantly higher in REAC TO-RGN treated as compared to untreated cells, just after 24 hours of exposure, and were retained higher even after 96 hours of treatment (Fig. 1). The same figure shows also that the expression of Nerve Growth factor (NGF), a known regulator of neuritogenesis in PC12 cells^7^ was significantly increased in REAC TO-RGN treated than in untreated cells, throughout the culturing period. The same figure shows that also the expression of tyrosine hydroxylase, a key enzyme in catecholamine biosynthesis was induced by REAC treatment (Fig. 1). Figure 2 shows the western blotting analysis of β3-tubulin, neurogenin 1, NGF and tyrosine hydroxylase, all of these proteins were significantly increased in REAC treated cells, confirming what previously observed by gene expression analysis (Fig. 1).

### REAC TO-RGN exposure induces the appearance of neuron-like cells

**Figure 3:**
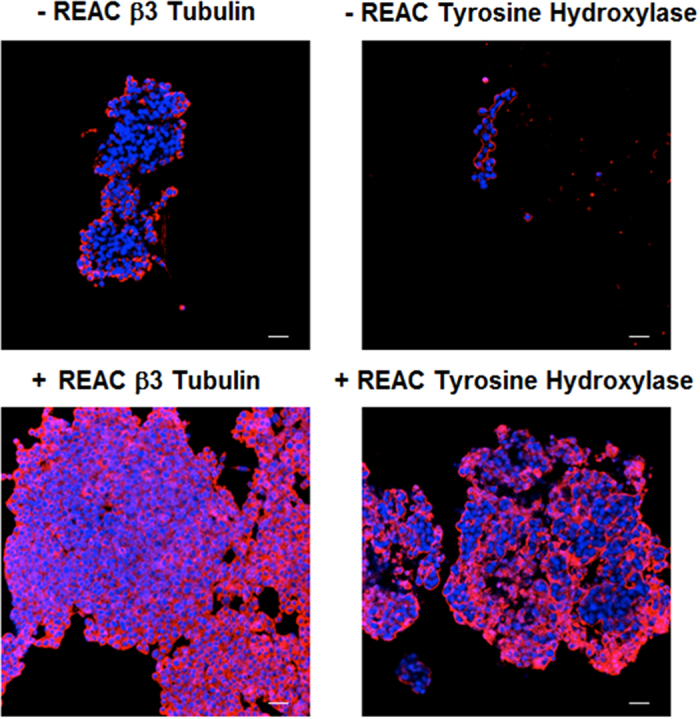
REAC TO-RGN induces PC12 differentiation toward neural phenotype. Expression of β-3-tubulin and Tyrosine Hydroxylase were assessed in cells cultured in the absence or presence of REAC TO-RGN treatment, for 168 h (7 days). Nuclei are labeled with DAPI (blue). Scale bars are 40 μm. Representative of five separate experiments. For each differentiation marker, fields with the highest yield of positively stained cells are shown.

Immunocytochemistry revealed that both untreated and REAC TO-RGN treated PC12 cells expressed the neuronal marker β-3 tubulin and the tyrosine hydroxylase, the rate-limiting enzyme of catecholamine biosynthesis, but in cells REAC TO-RGN treated both the number of positively stained cells, and the intensity of fluorescence, revealed a higher expression of both proteins, confirming what previously observed in β-3 tubulin gene expression analysis (Fig. 3).

### The effect of REAC TO-RGN on the proliferation of PC12 cells

**Figure 4:**
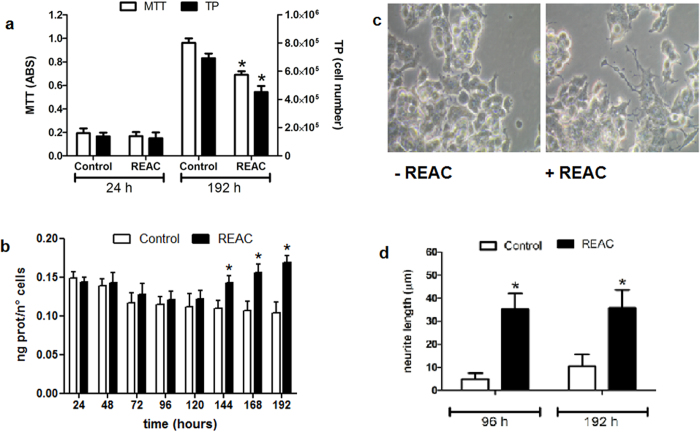
Panel a - MTT analysis and trypan blue staining and morphological changes in REAC TO-RGN treated PC12 cells. MTT (white bars) and Trypan blue (darker bars) cell proliferation analysis were performed 24 and 192 hours after culturing control and REAC TO-RGN treated cells. Panel b - Intracellular proteins quantification by Lowry method. It shows that the amount of total protein per cell is increased after the REAC TO-RGN treatment. Panel c - Microphotographies under a phase-contrast microscope show untreated cells (control) and REAC TO-RGN treated cells with neurites at 96 hours. Representative of five separate experiments. Panel d - Neurite length measurement. The morphological changing as neurite growth is shown in control and REAC TO-RGN treated cells after 96 and 192 hours.

Figure 4 (Panel a) shows both staining results from MTT assay and Trypan Blue assay at the beginning of the experiment (24 h) and at the end of it (192 h). At 24 h, no statistical differences in cell number were detected, neither in control cells nor in REAC TO-RGN treated cells. After 192 hours of treatment, REAC TO-RGN treated cultures displayed a significantly lower number of cells as compared to untreated cells. Measuring the protein content in controls and REAC TO-RGN treated cells, we found that there was no difference in the average content of proteins (data no shown), thus we calculated the amount of proteins per cell (Fig. 4, panel b). In control group, this ratio (protein content divided by cell number) showed a not statistically significant decrease during the first five days and then stabilized over the next three, when cells reached the confluence. In REAC TO-RGN exposed cells the amount of proteins per cell statistically increased of about 25% from the third to last day. This data suggest a higher level in protein synthesis and cellular differentiation, as shown by the increase in neurite length by the fourth day in treated cells compared to untreated cells. Indeed starting from the fourth day of treatment these cells have been shown cellular processes longer (P < 0.05) than those eventually present in the untreated cells (Fig. 4, panel c, d).

## Discussion

The use of PC12 cells as a model for the study of dopaminergic neurons, and also as a possible therapeutic tool in Parkinson’s disease^8^,^9^ has long been investigated. As known, the reduction of TH expression in the substantia nigra pars compacta of parkinsonian patients is a constant anatomo-pathological finding, while the striatal availability of DA is related to nigral TH expression. Despite the cell culture studies proposed in this manuscript are not conclusive for PD treatment and further studies are required in animal models of Parkinsonism (i.e. rats treated with MPTP or TAT-alpha-synuclein)^10^,^11^, the obtained data are suggestive of an enhancement of DA biosynthesis and availability. Studies are in progress for determining the effect of REAC treatment on PC12 cells DA biosynthesis and metabolism. Furthermore, the research for the use of physical means, in order to induce cell differentiation for new therapeutic strategies, is one of the most interesting challenges in the field of regenerative medicine, and then in the treatment of neurodegenerative diseases, Parkinson’s disease (PD) included. In this work, we have demonstrated that PC12 REAC TO-RGN exposure primes cell commitment toward a neurogenic phenotype and induce the appearance of neuron-like cells. Previous results clearly demonstrated that RGN treatment protocols was able to induce cell differentiation toward neural phenotype^1^,^2^,^3^. In the present study, we observed the increase in neuritogenesis, together with the significantly higher amount of protein synthesis in REAC TO-RGN treated cells as compared to control untreated. This discover is supported by the gene expression analysis, which highlights higher mRNA levels of neurogenin-1, an important transcription factor regulating neurogenesis^12^,^13^ and consequently of β3-tubulin, a neural phenotype specific marker^14^. The expression of this neural specific protein, as assessed by immunofluorescence and western blotting analysis, was also increased in REAC TO-RGN treated cells, confirming the gene expression analysis. These observations are further inferred by the increased expression of NGF observed in REAC TO-RGN exposed cells. Another interesting aspect revealed in this study, related to the increase of NGF, is the anti-proliferative effect in REAC TO-RGN -treated cells. NGF has been widely described as a regulator of PC12 proliferation leading to a decrease in proliferation rate, by inducing cell cycle arrest in G0/G1 phase^15^ and their terminal differentiation within the neural lineage^16^. Interestingly the analysis of cell viability clearly demonstrate an antiproliferative effect of REAC TO-RGN treatment on PC12 cells, while we previously demonstrated no viability reduction in human skin-derived fibroblasts^2^ and in human adipocyte derived stem cells^3^ exposed to REAC treatment. This event determinate an evident decrease in the number of PC12 tumor cells, presumably related to the NGF-induced neural differentiation. The data collected in this study, confirm the differentiation effect of the REAC TO-RGN treatments, previously observed in the induction of reprogramming and differentiation processes in normal cells^1^,^2^,^3^,^5^. Furthermore, the results obtained in this study, might suggest a possible future use of REAC treatments, as adjuvants in PD, even though further studies will be needed.

## Materials and Methods

### Reagents and solutions

All chemicals were analytical reagent grade and were used as supplied. Trypan Blue solution 0.4%, Thiazolyl Blue Tetrazolium Blue (MTT) powder, were purchased from Sigma (Milano, Italy). Dulbecco’s modified Eagle’s medium F12, horse serum (HS) and fetal bovine serum (FBS) and Streptomycin/Penicillin was bought by LIFE Technologies (Milano, Italy).

The phosphate-buffered saline (PBS) solution used for cell cultures was made using NaCl (137 mM), KCl (2.7 mM), Na2HPO4 (8.1 mM), KH2PO4 (1.47 mM), CaCl2 (1.19 mM), MgCl2 (0.54 mM), and glucose (7.5 mM) and then adjusted to pH 7.4: all reagents used for PBS solution were purchased from Sigma (Monza, Italy).

### Cell culture

PC12 cells were kept at 37 °C in 100 mm plastic culture plates in Dulbecco’s modified Eagle’s medium F12 (DMEM) enhanced with 10% HS, 5% FBS and a 1% of Streptomycin/Penicillin, in a humidified atmosphere of 5% CO_2_/ 95% air, as previously shown^17^. Cells were treated with REAC TO-RGN for 24, 48, 72, 96, 120, 144, 168 and 192 hours. Each sample was processed for MTT and Tripan Blue assay and for each sample protein content was evaluated by means of Lowry method^18^.

### Cell Viability Assays - MTT Assay

PC12 cells were treated or not (control) for 24 h and 8 days (192 h) with REAC TO-RGN in 24-well plates (60 × 10^3^ cells per well). Experiments were done in triplicate. Cell viability was assessed by the 3-(4, 5-dimethyl- thiazol-2-yl)-2, 5, diphenyltetrazolium bromide (MTT) assay^19^. In this assay, viable cells convert the soluble dye MTT to insoluble (in aqueous media) blue formazan crystals. At the beginning of each experiment, and at selected intervals, cell number was determined in triplicate wells. Briefly, 1 mg of MTT (200 μl of a 5 mg/ml stock solution in phosphate buffered saline (PBS)) was added per ml of medium and the cultures allowed to incubate at 37 °C or 4 h. The MTT was removed and the cells rinsed with PBS and centrifuged at 4000 rpm for 20 minutes. Thus the supernatant was discarded, the pellet dissolved in 2 ml isopropanol and after centrifugation at 4000 rpm for 5 min the color was read at 600 nm using a Bauty Diagnostic Microplate Reader. A standard curve was created utilizing different concentrations of cells at the beginning of every experiment. Before the experiments, PC12 cells were prepared in the cell laboratory of Pharmacology section and then transferred to the cell laboratory of Biochemistry. Only the treatment groups were subjected to REAC exposure. Subsequently, the PC12 cells were processed in both laboratories mentioned above by means of double-blind control procedures.

#### Trypan-Blue Staining

For each experiment, PC12 cells were exposed for 24 h and 192 h (8 days) to REAC TO-RGN in 24-well plates (60 × 10^3^ cells per well). Control groups were maintained in the same culture condition without REAC TO-RGN exposure. Experiments were done in triplicate.

For evaluating cell survival, the trypan blue (0.4%) exclusion assay, based on the capability of viable cells to exclude the dye, was used. As viable PC12 cells are capable of maintaining membrane integrity and preventing trypan blue dye to pass through the cell membrane, cells with damaged membrane appeared blue due to the accumulation of dye and were taken as dead cells. For trypan blue staining, 10 μl trypan blue solution and 90 μl of cells from each sample were incubated for 1 min. Unstained live cells were counted with Burker’s chamber.

### Description of Radio Electric Asymmetric Conveyer (REAC) technology

The REAC is an innovative-patented technology for bio-stimulation and/or bio-enhancement techniques. The REAC device generates an emission of microwaves of very weak intensity. The peculiarity of REAC technology is not the emission, but the particular physic link between the device and the patient’s body. The asymmetric conveyer-probe (ACP) represents this link. The tailored ACP applied on cell cultures or patient’s body makes possible create a radio electric circuit in combination with the local electrical environment of the cell cultures or tissues, to concentrate the induced radio electric microcurrents inner the cell cultures or tissues to be treated.

The ACP provides an electrical tension reference in the cell cultures or in the point of the body where it is applied and acts therefore as a kind of pole of attraction. The electrical tension reference is fixed and independent of the applied load (the characteristics of the tissues in treatment). For this phenomenon, the radio electric micro currents induced in the body are conveyed by the ACP in the point of application. Thanks to the radio electric microcurrents asymmetrically conveyed in the body, REAC technology is able to normalize currents flows existing in the cell cultures or human body, when these were reduced in quantity and altered in the transmission within the tissues. The REAC electromagnetic quantities have been measured with the spectrum analyzer Tektronix model 2754p (TekNet Electronics, Inc., Alpharetta, GA, USA), orienting the receiving antenna for maximum signal. With cell cultures at a distance of 35 cm from the 2.4 GHz. emitter, we measured a radiated power of approximately 400 μW/m2. Electric field E = 0.4 V/m, magnetic field = 1 mA/m. Specific absorption rate (SAR) = 0.128 μW/g.

### REAC tissue optimization - regenerative treatments (TO-RGN)

REAC tissue optimization (TO) regenerative treatments (RGN) includes a set of treatment protocols^1^,^2^,^3^,^4^,^5^,^20^ which are carried out by the ACP immersed in the medium of the cell culture or covering the treated area with ACP. The REAC TO-RGN has been administered 168 hours without interruption. The REAC device used in this study (B.E.N.E Bio enhancer - Neuro enhancer, ASMED, Florence, Italy) is specific for regenerative treatments.

### Cell Differentiation

PC12 cells (10^5^ cells/mL) were differentiated by REAC TO-RGN exposure as previously described^2^. Briefly, PC12 cells growing in 100-mm culture dishes were treated with REAC TO-RGN for 24, 48, 72, 96, 120, 144, 168, and 192.

### Morphological analysis

Microphotographs of PC12 cells from untreated cultures (control) and from treated ones were taken after 96 hours of REAC TO-RGN treatment to track any morphologic changes. Collected images were loaded into ImageJ (National Institute of Health, USA) and analyzed to measure neurite length.

### Gene expression analysis

**Figure 5:**
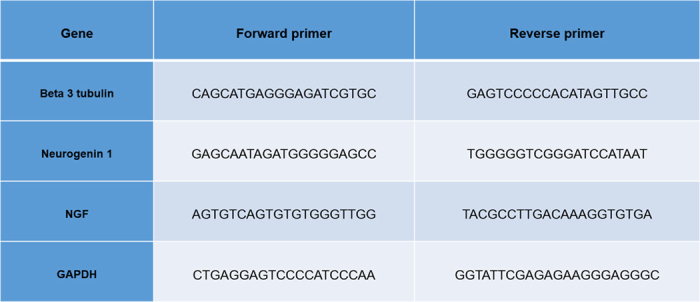
Primers used for the analysis of neurogenesis (Neurogenin-1 and beta 3 tubulin) and nerve growth factor (NGF).

PC12 cells were cultured in the absence or presence of REAC TO-RGN for 24, 48, 72, 96, 120, 144, 168, 196 hours. Total RNA was isolated using Trizol reagent according to the manufacturer’s instructions (Invitrogen). Total RNA was dissolved in RNAase-free water and, for RT- PCR, cDNA was synthesized in a 50-μl reaction volume with 1 μg of total RNA using Superscript Vilo cDNA synthesis kit, according to the manufacturer’s instruction (Life Technologies). Quantitative real-time PCR was performed using an iCycler Thermal Cycler (Bio-Rad). Two μl of cDNA were amplified in 50-μl reactions using Platinum Supermix UDG (Invitrogen), 200 nM of each primer, 10 nM fluorescein (BioRad), and SYBR Green. After an initial denaturation step at 94 °C for 10 min, temperature cycling was initiated. Each cycle consisted of 94 °C for 15 s, 55– 59 °C for 30 s, and 60 °C for 30 s, the fluorescence being read at the end of this step. Primers used for the analysis of neurogenesis (Neurogenin-1 and beta 3 tubulin) and nerve growth factor (NGF) were specific and spanning exons, are reported in Fig. 5.

To evaluate the quality of product of real-time PCR assays, melting curve analysis was per-formed after each assay. Relative expression was determined using the “delta-CT method” with glyceraldehyde 3-phosphate dehydrogenase (GAPDH) as reference gene^21^.

### Immunocytochemistry

Cells were cultured for 7 days with or without REAC TO-RGN treatment on glass coverslips coated with 0.2% type III Collagen at low density to permit visualization of individual cells. After 7 days, cells were fixed for 20 min in 4% paraformaldehyde (Life Technologies). The cells were washed 3 times with phosphate-buffered saline (PBS) with 0.1% Triton X-100, and then incubated with the primary antibody overnight at 4 °C. The cells were then incubated with the secondary antibody. To verify differentiation toward a dopaminergic neuronal phenotype, the following antibodies were used: mouse monoclonal anti-ß-3 tubulin (Santa Cruz Biotechnology); rabbit polyclonal anti- tyrosine hydroxylase (Santa Cruz Biotechnology). The secondary antibodies used in this study were: Alexa 594-conjugated goat-anti-rabbit IgG (1:400; Life Technologies) and anti-Mouse IgG (whole molecule) −TRITC antibody produced in goat (Sigma-Aldrich). All microscopy was performed with a Leica confocal microscope (LEICA TCSSP5). DNA was visualized with 1μg/ml 4’,6-diamidino-2-phenylindole (DAPI).

### Western blotting

Cells were cultured in the presence or absence of REAC for 24, 48, or 72 hours and for 7 days. Total cell lysates from PC12 were electrophoresed on 10% Novex Tris-glycine polyacrylamide gels (Invitrogen) in MOPS sodium dodecyl sulfate running buffer using an XCell SureLock™ Mini-Cell (Invitrogen) according to the instructions provided by the manufacturer. After protein transfer to polyvinylidenedifluoride membranes (Invitrogen), and membrane saturation and washing, immunoreaction was carried out for one hour at room temperature in the presence of the primary antibody, antisera against β Tubulin isotype III (Cell Signaling technology), NGF (Santa Cruz Biotechnology, Inc.), TH (Santa Cruz Biotechnology, Inc), Neurogenin (Santa Cruz Biotechnology, Inc), and GAPDH (Santa Cruz Biotechnology, Inc.) diluted to 1:1000. After additional washing, membranes were incubated with antirabbit (NGF ,GAPDH) or antimouse (β Tubulin isotype III) or antigoat (Neurogenin). Targeted protein expression was assessed using a chemoluminescence detection system (ECL Western blotting detection reagents were from Amersham Biosciences Corporation, Piscataway, NJ, USA).

### Statistical analysis

Statistical analysis was performed using the software package SPSS (Statistical Package for Social Science) version 13.0. For this study, were applied to the data collected, the nonparametric Kruskal-Wallis test, Jonckheere-Terpstra and Wilcoxon test: the first two to assess the adequacy of treatment, i.e. the differences (DeltaDelta CT) between the data collected in treated and in control. The Wilcoxon test was applied to assess the adequacy of the data of each group in the different observation times. The tests and results with p < 0.05 were considered statistically significant. For the adequacy of the data, the results of the Wilcoxon test showed a high statistical significance in all observations (Asymp. Mr. 2-tailed < 0.05). For the congruity of the treatments, the tests were applied to 3 sets, in eight moments of observation, after the treatment and control conditions. The analysis shows a statistical significance p < 0.05 in the treated (Kruskal-Wallis test: Asymp. Sig. 2-tailed < 0.417 and Jonckheere-Terpstra test: Asymp. Sig. 2-tailed < 0.295) and p > 0.05 in controls. (Kruskal-Wallis test: Asymp. Sig. 2-tailed = 0.7 and Jonckheere-Terpstra test: Asymp. Sig. 2-tailed > 0.75).

## Author Contributions

S.R. and V.F. invented REAC technology, collaborated in conceiving the experimental design and wrote the manuscript. M.M., P.A.S. and R.M. conceived and designed the experimental plan and wrote the manuscript. G.P., G.R., S.S., A.C. and V.B. performed the experiments and prepared figures. C.V. supervised the project. All authors reviewed the manuscript.

## Additional Information

**How to cite this article**: Maioli, M. *et al.* Neurological morphofunctional differentiation induced by REAC technology in PC12. A neuro protective model for Parkinson's disease. *Sci. Rep.* **5**, 10439; doi: 10.1038/srep10439 (2015).
